# Influence of Vitamin C on Lymphocytes: An Overview

**DOI:** 10.3390/antiox7030041

**Published:** 2018-03-10

**Authors:** Gwendolyn N. Y. van Gorkom, Roel G. J. Klein Wolterink, Catharina H. M. J. Van Elssen, Lotte Wieten, Wilfred T. V. Germeraad, Gerard M. J. Bos

**Affiliations:** 1 Division of Hematology, Department of Internal Medicine, GROW-School for Oncology and Developmental Biology, Maastricht University Medical Center, 6202AZ Maastricht, The Netherlands; r.kleinwolterink@maastrichtuniversity.nl (R.G.J.K.W.); janine.van.elssen@mumc.nl (C.H.M.J.V.E.); w.germeraad@maastrichtuniversity.nl (W.T.V.G.); gerard.bos@mumc.nl (G.M.J.B.); 2 Department of Transplantation Immunology, Maastricht University Medical Center, 6202 AZ Maastricht, The Netherlands; Lotte.wieten@maastrichtuniversity.nl

**Keywords:** vitamin C, ascorbic acid, lymphocytes, natural killer cells, NK cells, B cells, T cells

## Abstract

Vitamin C or ascorbic acid (AA) is implicated in many biological processes and has been proposed as a supplement for various conditions, including cancer. In this review, we discuss the effects of AA on the development and function of lymphocytes. This is important in the light of cancer treatment, as the immune system needs to regenerate following chemotherapy or stem cell transplantation, while cancer patients are often AA-deficient. We focus on lymphocytes, as these white blood cells are the slowest to restore, rendering patients susceptible to often lethal infections. T lymphocytes mediate cellular immunity and have been most extensively studied in the context of AA biology. In vitro studies demonstrate that T cell development requires AA, while AA also enhances T cell proliferation and may influence T cell function. There are limited and opposing data on the effects of AA on B lymphocytes that mediate humoral immunity. However, AA enhances the proliferation of NK cells, a group of cytotoxic innate lymphocytes. The influence of AA on natural killer (NK) cell function is less clear. In summary, an increasing body of evidence indicates that AA positively influences lymphocyte development and function. Since AA is a safe and cheap nutritional supplement, it is worthwhile to further explore its potential benefits for immune reconstitution of cancer patients treated with immunotoxic drugs.

## 1. Introduction

Vitamin C or ascorbic acid (AA) has often been linked to cancer treatment. Already in the 1970s, Cameron and Pauling reported that high doses of AA intravenously increased the survival time of terminal cancer patients more than four times [1] but this finding could not be repeated in other studies where AA supplementation was given orally [2,3]. However, subsequent studies show that AA has a wide variety of effects on cancer cells and the immune system. In this review, we discuss the effects of AA on lymphocytes in the light of cancer treatment.

AA is an essential micronutrient for humans with many functions in the human body. It is an antioxidant and a free radical scavenger and serves as an essential cofactor for many enzymatic reactions through iron-, copper- and 2-oxoglutarate-dependent dioxygenases. Among many other functions, these dioxygenases are important in epigenetic regulation by catalysing the hydroxylation of methylated nucleic acids (DNA and RNA) and histones [4]. While most mammals use the enzyme gulono-gamma-lactone oxidase to synthesize AA in the liver, many primates and humans carry a non-functional copy of the *GULO*-gene and consequently depend on dietary sources of AA. When studying the effects of AA and AA deficiency in vivo in animal models, this is a complicating factor. Guinea pigs, like humans, also have a defect in the GULO-gene and are thereby often chosen for AA deficiency studies. Alternatively, there are two knockout mouse models, a *Gulo* knockout (*Gulo*^−^/^−^) and a senescence marker protein-30 knockout (*SMP30KO*), in which biosynthesis of AA in the liver is blocked [5,6].

AA has an extensive role in the immune system. Its role in phagocytic cells like neutrophils, has been investigated thoroughly and was recently reviewed [7]. In summary, AA enhances chemotaxis and phagocytosis of phagocytes and thereby promotes microbial killing. In contrast, the role of AA in different subsets of lymphocytes is less clear. Since lymphocytes actively acquire AA via sodium-dependent vitamin C transporters (SVCT) and sodium independent glucose transporters (GLUT) (reviewed in [8] and have intracellular AA concentrations that are 10–100-fold higher than plasma levels [9,10], it is likely that AA has an essential function in these cells. There are three main subsets of lymphocytes, namely T cells, B cells and natural killer (NK cells). T cells are involved in cell-mediated, cytotoxic adaptive immunity, B cells are responsible for the adaptive, humoral immunity and NK cells are part of the innate, antigen-independent immunity.

In our laboratory, we are interested in lymphocytes in cancer treatment, because these cells are often destroyed by anticancer treatment and take time to recover. During this phase, patients are highly susceptible to possibly lethal infections. Depending on the intensity of the chemotherapy used, this period may be relatively short, for example in breast cancer, or long, for example in leukaemia. After so-called myeloablative chemotherapy, hematopoietic stem cells (HSC) that are located in the bone marrow have to be replaced in order to restore all types of blood cells, including leukocytes. In particular, the regeneration of T-lymphocytes, a subset of lymphocytes that are especially important to fight against viral infections, is slow as a consequence of age-dependent involution of the thymus, the organ that is required for their development [11,12]. Looking at ways to improve T cell recovery after cancer therapy, we investigated factors that influence human T-lymphocyte development and found that AA acts as a factor that promotes maturation of T cells. AA is also indispensable for T cell development in vitro [13]. Additionally, we showed that NK cells regenerate faster under the influence of AA [14].

We also found that haematological cancer patients often have severely decreased serum AA levels compared to healthy controls (20.5 ± 12 μM versus 65 ± 4 μM, respectively). Serum AA levels were even undetectable in 19% of patients with a haematological malignancy, irrespective of the choice of treatment [15]. Since AA is a cheap and readily available supplement with a safe profile, it is attractive to speculate that cancer patients who need to regenerate their immune system after chemotherapy with or without hematopoietic stem cell transplantation (HSCT) may benefit from the effects of AA on immune reconstitution. In this way, we hypothesize that mortality and morbidity resulting from opportunistic infections could be reduced. It could also be that NK cells regenerate faster and are able to kill cancer cells sooner. AA supplementation could also be used in cellular therapies, where in vitro proliferated and adapted subtypes of lymphocytes are used to eliminate tumour cells in vivo. However, before using AA in clinical applications, it is important to have a better understanding of the role of AA in these lymphocytes.

In this article, we highlight the effects of AA on different subsets of lymphocytes as far as they are known for this moment. We will focus on the effects on the physiology of these cells and on the role of AA on lymphocytes in health and disease and not on the potential mechanisms behind these effects, since this was extensively reviewed before [4].

## 2. AA and T Lymphocytes

T lymphocytes are a major component of the human immune system and are involved in cell-mediated, cytotoxic adaptive immunity. On their surface, T cells express the T cell receptor (TCR) that is responsible for recognizing and binding specific antigens bound to major histocompatibility complex (MHC) molecules. There are different types of T lymphocytes, including cytotoxic T cells, T helper cells, memory T cells and regulatory T cells. Cytotoxic T cells are characterized by a MHC class I binding CD8 protein on their cell surface. The TCR and CD8 receptor bind infected cells and tumour cells. After binding, the cytotoxic T cells mature and, upon activation by an infected cell, secrete perforin and granzymes, that kill the infected cells. T helper cells are CD4 positive cells that regulate immune responses. Their TCR binds to MHC class II on antigen presenting cells (APCs). After binding, T-helper cells secrete cytokines that activate other immune cells, including cytotoxic T cells. Memory T cells are long-living cells that recognize previously encountered pathogens and provide lifelong immunity. Regulatory T cells shut down T cell mediated immunity toward the end of an immune reaction and help to maintain tolerance to self-antigens.

Here we describe the effects on AA on general T cell development and summarize what is known about the influence of AA on these most important subsets of T cells. We will not discuss cytotoxic T cells since we found no studies examining the effects of AA on this specific subset.

### 2.1. T Cell Development and AA

T cell development is a tightly controlled process that takes place in the thymus, which can be simulated in vitro using foetal thymic organ cultures [16], stromal cells [17] or in feeder-free conditions [13]. While mature T lymphocytes express either CD4 or CD8 for helper and cytotoxic subsets respectively, immature T cells are called “double negative” (DN) because they lack CD4 and CD8 expression. Traveling through the highly-organized thymus, the developing T cells undergo numerous rounds of proliferation. The thymic stromal cells provide the structural support and cytokines necessary for selection of a functional TCR that does not recognize self-antigens. This process of “education” is required to generate a diverse repertoire of TCRs to ensure immunity against a wide variety of antigens. The various stages of human T cell development are characterized by sequential acquisition of CD7, CD5, intracellular CD3, CD1a, CD4 and CD8, TCRαβ and surface CD3 [18].

In search for factors that enhance T cell differentiation after stem cell transplantation, we discovered that AA enhances human T cell proliferation in vitro [13]. Beside this effect on T cell proliferation, we also found multiple effects on early T cell development. Most importantly, we showed that AA is required in vitro for the transition of DN precursors to the next, so-called “double positive” (DP, CD4^+^ CD8^+^) stage in feeder-free cultures as well as in cultures with stromal cells when culturing T cells from cord blood or G-CSF stimulated hematopoietic stem cells. Furthermore, we found that in a feeder-free system, early maturation of T cells after 3 weeks was improved under the influence of AA in a dose dependent way with an optimum at 95 µM [13]. These results are in line with a murine study in which the investigators cultured adult bone marrow-derived hematopoietic progenitor cells on stromal cells and showed that these cells only differentiate to the DP stage in the presence of AA. To determine the effect of AA in vivo, foetal liver chimeric mice were generated by transfer of *Slc23a2*-deficient HCS into recipient mice. In the absence of *Slc23a2*, hematopoietic cells are unable to concentrate AA. Consequently, in animals with a *Slc23a2*-deficient hematopoietic system, T cell maturation was virtually absent compared to control mice [19].

Since AA functions as an antioxidant, we tested if other antioxidants could restore T cell development. As this was not the case, the effect of AA on developing human T cells cannot be attributed to its antioxidant properties [11]. This finding is supported by Manning et al. [19] who showed that induction and maintenance of *Cd8a* gene expression is dependent on AA-dependent removal of repressive histone modifications, rather than on its function as an antioxidant.

In summary, in humans and mice, AA is required in vitro for the early development of T cells as it overcomes a development block from DN to DP. Furthermore, AA speeds up the maturation process of T lymphocytes. In mice, at least part of this effect is due to AA-dependent epigenetic regulation.

### 2.2. T Cell Proliferation and AA

Multiple researchers studied the effect of vitamin C on the proliferation and survival of T cells, in vitro as well as in vivo.

One study describes the effect of AA on in vitro culture of in vivo activated mouse T cells. While more than 70% apoptotic cells were found in cultures without AA, the addition of AA (450 µM) decreased apoptosis by one-third and induced more proliferation was seen compared to cultures without AA [20]. In another study, evaluating the effects of AA on murine T cells during in vitro activation, it was found that that low concentrations (62.5 μM and 125 μM) of AA do not change proliferation or viability of T cells, while higher concentrations (250 μM and 500 μM) do decrease both [21]. In a third study, researchers examined how AA prevents oxidative damage using purified human T cells. They report similar effects: medium-high concentrations of AA (57–142 μM) decrease T cell proliferation, while at higher concentrations (284 μM), AA decreases cell viability and IL-2 secretion more than 90% [22]. In another study studying the expression of SVCT on T cells, the investigators show a similar effect. Peripheral blood T cells of healthy human volunteers were activated in vitro in the absence or presence of different doses AA, before and after activation. AA did not have any effect on proliferation or apoptosis in low doses (62.5–250 μM). At high doses (500–1000 μM), the proliferation was inhibited and there was an increase in apoptosis when AA was added before activation [23].

In a study on the effect of AA-deficiency on lymphocyte numbers in guinea pigs, the investigators found that in animals with an 4-week AA-free diet, the number of T-lymphocytes decreased continuously while T cell number slightly increased in AA-supplemented animals (25 and 250 mg intraperitoneally/day) [24]. Plasma and tissue concentrations of AA were significantly lower in animals without AA compared to AA-treated animals. In another in vivo study using AA-deficient *SMP30KO*^−/−^ mice, the researchers determined the long-term effect of AA on immune cells using a diet with an increased AA level (200 mg/kg vs. 20 mg/kg). During the one-year study, T-lymphocytes in the peripheral blood increased in number. More specifically, the number of naive T cells, memory T cells in the spleen and mature T cells in the thymus [6] increased. Plasma concentrations of AA in mice with a low-dose AA diet were similar to wildtype mice, while plasma concentrations in the high-dose diet were significantly higher.

Badr et al., examined if the impaired T cell function in type I diabetes can be improved by AA supplementation using a streptozotocin-induced diabetes type I rat model. These animals have diminished T cell cytokine production, less proliferation and lower surface expression of CD28, a protein that is important for T cell activation and survival. AA supplementation (100 mg/kg/day for 2 months) restored the CD28 expression, cytokine secretion and proliferation [25].

Studies in humans are limited. Because elderly people often have lower serum levels of AA and are more prone to infections, a placebo-controlled trial was performed in which elderly people received either an intramuscular injection of AA (500 mg/day) or placebo for 1 month. Compared to the placebo group, an increase in T cell proliferation was seen in the AA-supplemented group [26]. The only other study in humans could not recapitulate this effect [27]. Healthy volunteers were kept on an AA-free diet during a 5-week period to induce AA deficiency. This did not lead to any changes in T cell numbers or function, while the induction of AA deficiency was confirmed in plasma and leukocytes.

In summary, both animal and human studies show that physiological AA concentrations have a beneficial effect on T cell proliferation, while supraphysiological concentrations are toxic for T cells. In vivo, restoration of AA in deficient patients positively influences T cell proliferation as well, while this observation could not be reproduced in induced AA-deficiency.

### 2.3. T Helper Cells (Th) and AA

There are several subsets of Th cells, the most important ones being Th1, Th2 and Th17. Th1 cells are part of the defence against intracellular bacteria and protozoa. Using their main effector cytokines IFN-γ and TNF-α, they activate cytotoxic T cells and macrophages. Th2 cells are effective against extracellular parasites and produce mainly IL-4, IL-5 and IL-13. They stimulate eosinophils, basophils, mast cells and B-cells. Th2 cells are important mediators of allergy and hypersensitivity. For this reason, Th2 cells are often investigated in animal models for asthma. Th17 cells have an important role in pathogen clearance of mucosal surfaces and produce IL-17, a cytokine that stimulates B-cells. The various Th subsets differentiate from naïve CD4^+^ T cells in a process called “polarization”. In vivo, dendritic cells (DC) are the most important antigen-presenting cells (APC) that steer Th polarization via the production of cytokines.

Several researchers report that AA induces a shift of immune responses from Th2 to Th1. In one of these studies, a mouse model was used to examine the effect of AA (5 mg/day) on delayed-type hypersensitivity response against 2,4,-dinitro-I-fluorobenzene (DNFB). In this study, mice were intraperitoneally injected with AA before, during or after sensitization with DNFB. If T cells of mice supplemented with AA during the sensitization were later stimulated ex vivo, higher levels of Th1 cytokines (TNF-α and IFN-γ) and lower levels of Th2 cytokines (IL-4) were observed. This effect was not observed when mice were supplemented with AA before or after sensitization [28]. This modulation of immune balance from Th2 to Th1 was also seen in another study, in which the effects of AA supplementation on asthma was studied. Here, AA supplementation (130 mg/kg/day for 5 weeks) of ovalbumin-sensitized mice significantly increased the IFN-γ/IL-5 secretion ratio in bronchoalveolar lavage fluid compared to control mice, confirming a shift from Th2 to Th1 [29]. The mechanism underpinning this effect has not been elucidated yet but it was suggested to be mediated by DCs. In an in vitro study, murine bone marrow-derived DCs were pre-treated with different doses of AA before being activated with lipopolysaccharide (LPS). The DCs that were treated with AA secreted more IL-12, a polarizing cytokine for Th1 cells. It also showed that naïve murine T cells, when co-cultured with these activated and AA treated murine DCs, produced more IFN-γ and less IL-5 verifying this effect [30].

While most studies focus on the Th1/Th2 balance, we found only one study that describes that Th17 polarization of sorted murine naïve CD4^+^ cells is more effective in the presence of AA [31]. Interestingly, the investigators demonstrate that this effect is probably due to AA-mediated effects on histone demethylation that enhances the expression of the IL-17 locus.

In summary, multiple animal studies show that AA promotes Th1 differentiation at the expense of Th2 polarization. There is limited data showing that Th17 polarization is promoted by AA acting as an epigenetic regulator.

### 2.4. Memory T Cells and AA

Memory T cells constitute a small subset of lymphocytes but provide life-long immunity to previously encountered antigens. At this moment, the effects of AA on memory T cells is hardly investigated. Jeong et al. examined the effect of DCs pre-treated with AA on CD8^+^ T cell differentiation. In vitro, murine bone marrow-derived DCs were pre-treated with AA before activation with LPS and, like in the earlier study from the same research group [30], secreted more IL12p70 but also more IL-15, a cytokine that is linked to memory T cell generation. These DCs in co-culture with murine T cells led to an enhanced CD8^+^ memory T cell production. The effect was also seen in a mouse model for melanoma, in which the immune response and anti-melanoma effect of melanoma-primed DCs was enhanced if pre-treated with AA: the investigators observed increased generation of tumour-specific CD8^+^ memory T cells and an increased protective effect for inoculated melanoma cells [32].

In summary, in vitro and in a mouse model AA increased the generation of CD8^+^ memory T cells through increased production of stimulating cytokines by DCs.

### 2.5. Regulatory T Cells (Tregs) and AA

Tregs are important for the maintenance of immune-balance and self-tolerance. They are characterized by the expression of the transcription factor Foxp3, required for their immunosuppressive capacity. Stable expression of Foxp3 is dependent on DNA demethylation of a region in the first intron. Two recent in vivo and in vitro studies on murine Tregs found that AA stabilizes expression of Foxp3 by promoting active Ten Eleven Translocation (TET) 2-mediated DNA methylation of this region. Thus, AA is required for the development and function of Tregs [33,34]. Concordantly, AA is a known co-factor for Ten Eleven Translocation (TET) family proteins that catalyse the first step of DNA demethylation: the conversion of 5-methyl-cytosine (5 mC) to 5-hydroxy-methyl-cytosine (5 hmC) [35,36]. For instance, in embryonic stem cells, AA was shown to be an important epigenetic regulator through this pathway [37]. The addition of AA to embryonic stem cell cultures induced demethylation of over 2000 genes within one hour [38].

In another study, the influence of AA on skin graft rejection in mice after treatment with ex vivo cultured and alloantigen-induced Tregs was investigated. The in vivo alloantigen-induced murine Tregs showed more DNA demethylation and stability of Foxp3 expression when cultured in the presence of AA. These Tregs also showed better suppressive capacity in vivo, thereby promoting skin allograft acceptance [39]. In a mouse model for graft-versus-host-disease (GVHD), the effects of AA on these ex vivo alloantigen-induced Tregs was determined [40]. GVHD is a serious and sometimes lethal complication following allogeneic hematopoietic stem cell transplantation caused by alloreactive donor T cells that induce tissue injury in the recipient. In this model, in vitro murine alloantigen-induced Tregs pre-treated with AA showed more stable Foxp3 expression when transferred into mice with acute GVHD and were clinically effective to diminish GVHD symptoms. Moreover, cultured human alloantigen-induced Tregs also had a higher Foxp3 expression if cultured with AA.

In contrast, in a mouse model for sepsis, AA was found to decrease the inhibition of Tregs [41]. Here, sepsis was induced in AA-deficient *Gulo*^−^/^−^ mice that were supplemented with AA (200 mg/kg twice) or not. AA administration improved survival in both wild type and *Gulo*^−^/^−^ mice and diminished the negative inhibition of Tregs by decreasing the expression of Foxp-3, CTLA-4, a protein that functions as an immune checkpoint and downregulates immune responses and the inhibitory cytokine TGF-β.

In summary, AA directly regulates Treg function via epigenetic regulation of the master transcription factor Foxp3. In most studies employing more chronic situations (transplantation, GVHD), Foxp3 expression is increased. In this way, AA can be useful in generating ex vivo allo-antigen-induced Tregs that can be used for clinical applications in transplantation and autoimmune disorders. In one model of acute sepsis in mice, AA administration decreased Foxp3 expression but was still beneficial for the outcome.

## 3. AA and B Lymphocytes

B lymphocytes are at the centre of the adaptive, humoral immune system. They are responsible for the production of antigen-specific immunoglobulin (Ig) directed against invasive pathogens (antibodies). Similar to other leukocytes, AA accumulated in B lymphocytes but there is only limited data on the function of AA in these cells.

### 3.1. B Lymphocyte Numbers and AA

In an early study investigating the effect of AA deficiency on numbers of lymphocytes in guinea pigs, animals on a 4-week AA-free diet showed a continuous increase in the percentage of B-lymphocytes while the percentage of T-lymphocytes decreased. The opposite effect was seen in animals on AA supplementation (25 and 250 mg intraperitoneally/day) [24]. In a more recent and extensive study, the effect of AA-2G, a stable vitamin C derivate, was investigated on mouse B cells in vitro. Mouse spleen B cells were cultured for 2 days with an anti-μ antibody in the presence of stimulating cytokines and then washed and recultured with and without AA-2G. In these cultures, the number of viable cells decreased much quicker without AA, resulting in about 70% more viable cells in cultures with AA than without AA. AA-2G also increased the production of IgM dose-dependently [42]. Another group that studied the effect of AA on mouse spleen B cells in vitro published contradictive results. They showed a slight dose-dependent increase of apoptosis (16% at a concentration of 1 mM) in murine IgM/CD40-activated B cells pre-treated with AA [43]. Tanaka et al. investigated the effect of AA on immune responses in human peripheral blood lymphocytes cultured for 7 days with and without AA-2G before stimulating them with pokeweed mitogen (PWM), a T cell dependent B cell stimulus. The cultures treated with AAS-2G showed an increased number of IgM and IgG-secreting cells after stimulation [44].

### 3.2. B Lymphocyte Function and AA

We only found limited and conflicting data on the effect of vitamin C on the production of antibodies by B lymphocytes. Two early studies in guinea pigs show that high-dose AA supplementation increases immunoglobulin levels after immunization with sheep red blood cells (SRBC) and bovine serum albumin (BSA) [45,46]. However, in two other animal models, AA supplementation did not have an effect on antigen-induced immunoglobulin levels after immunization [47,48]. One study determined the effect of high dose AA (2500 mg/day for 4 weeks) in mice sensitized by topical application of di-nitro-chlorobenzene (DNCB) and re-challenged 2 weeks later. They found no effect of AA on immunoglobulin levels [47]. In the other study, the effect of different low doses of AA (0, 30 and 60 mg) on the immune functions of dogs immunized with PWM was studied but no differences in PWM-specific IgG and IgA levels were observed [48]. However, the latter two studies were performed in animals that are able to synthetize AA, while guinea pigs cannot. In AA-synthetizing calves, AA supplementation (1.75 g/day) led to lower plasma IgG levels against keyhole limpet haemocyanin (KLH), a T cell dependent antigen that is often used in immunological studies in animals [49]. Two studies were performed in AA-sufficient chickens to investigate if AA supplementation is beneficial for vaccination against infectious bursal disease (IBD). Chickens with AA supplementation (1 g/mL) showed higher immunoglobulin levels compared to chickens without extra AA [50,51]. Furthermore, non-vaccinated chickens receiving AA supplementation did not show any symptoms or mortality after challenge with IBD while non-vaccinated chickens without AA all experienced clinical symptoms and only 70% survived [50].

In one study in healthy human volunteers, researchers found a correlation between serum IgG and plasma and leukocyte AA concentration and serum IgM and leukocyte AA concentration. After daily supplementation of 1 g AA during one week, serum IgG significantly rose in those healthy volunteers as did plasma and leukocyte AA concentration [52]. Likewise, another study in healthy human volunteers showed an increase in serum levels of IgM and IgA after 1 g/day supplementation of AA for 75 days [53]. These findings were contradicted in two other studies. In one study, the investigators examined the effect of 2–3 g AA supplementation per day on the production of all immunoglobins in healthy volunteers and did not find any change [54]. The other study is an earlier described placebo-controlled trial in elderly people where they received either an intramuscular injection of AA (500 mg/day) or a placebo for 1 month. Also in this trial, AA supplementation did not have any influence on serum IgA, IgM and IgG levels [26].

In conclusion, it is possible that vitamin C has an effect on the proliferation and function of B lymphocytes but the results until now are inconclusive. When conducting an intervention study of AA in cancer patients it would be interesting to also examine B cell levels and immunoglobulin changes.

## 4. AA and Natural Killer Cells

Next to B and T lymphocytes, NK cells are the most prominent lymphocyte subset as they make up to 20% of the blood lymphocyte population and are important for the immunity against pathogens (especially viruses) and for tumour surveillance. They are large granular lymphocytes arising from the same lymphoid progenitors as T and B lymphocytes and are primarily formed in the bone marrow. NK cells are innate lymphoid cells (ILCs) that provide fast, antigen-independent immunity. They exhibit direct cytotoxic effects, secrete cytokines and chemokines and regulate other immune cells. NK cell cytotoxicity is based on the absence of self MHC class I to discriminate between normal and diseased cells. Killing of these MHC class I missing target cells can only be initiated after simultaneous detection of activating signals, like stress signals, on the surface of tumour or infected cells.

Our knowledge about the effects of AA on NK cell development is limited. We previously described that AA (95 µM) enhances proliferation of mature NK cells from peripheral blood mononuclear cells (PBMCs) in vitro in a cytokine-stimulated culture [14]. AA also improved the generation and expansion of NK cell progenitors from hematopoietic stem cells and from T/NK cell progenitors in vitro in a cytokine-stimulated culture.

Other studies investigated the role of AA on the function of NK cells. In our previously described study, we tested functionality of the mature NK cells that were expanded in vitro in a cytotoxicity assay on K562 cells, a chronic myeloid leukaemia cell line that is often used to assess NK cell function in vitro. There was no difference in killing capacity between NK cells that were cultured with or without AA. [14]. In contrast, an earlier study on the cytotoxicity of fresh human NK cells isolated from peripheral blood that had different doses of AA (10 µM to 2.5 mM) present during the killing showed a dose-dependent decrease of NK cell mediated killing of K562 cells in vitro [55]. A similar experiment was repeated in another study but with different results. A presence of 3 mM AA increased the cytotoxicity of NK cells 105% in average, while no change was found using lower concentrations (10 µM, 0.1 mM and 1 mM) [56].

Another group also investigated the effect of AA on peripheral blood NK cell function using a cytotoxicity assay on K562 with NK cells from healthy volunteers that were supplemented with a single high dose of vitamin C. The study showed a biphasic effect on NK cell cytotoxicity: a slight decrease 1 to 2 h after supplementation followed by a significant enhancement at 8 h with a maximum effect after 24 h and return to normal after 48 h [57]. Plasma and leukocyte concentration of AA increased after 1 h and maximized after 2 to 4 h. After that, the levels declined but were still elevated up to 24 h after supplementation. Since NK cell function is often decreased after exposure to toxic chemicals, these researchers also performed a similar experiment in 55 patients that where accidently exposed to various toxic chemicals (for instance pesticides, metals and organic solvents). Almost half of these patients showed a very low baseline NK cell activity and in 78% of the patients there was significantly enhancement of cytotoxicity compared to baseline 24 h after ingestion of 60 mg/kg AA [58]. It is difficult to interpret these findings, since the patients were very diverse and there was no control group.

A comparable study was performed with NK cells isolated from peripheral blood of patients with β-thalassemia major. NK cells of these patients show a severe reduction in their cytotoxic function compared to healthy controls, possibly due to oxidative stress caused by iron overload after multiple blood transfusions [59]. AA (200 µg/mL) almost normalized the cytotoxic capacity of these NK cells, while the NK cell function of healthy controls did not change [60]. Remarkably, AA and iron are connected in many biological processes. For example, AA enhances the absorption of nonheme iron from the intestines [61] and AA is an essential cofactor in many enzymatic reaction by iron-dependent dioxygenases. The positive effect of AA on the NK cell function in this case is probably related to its antioxidant properties, since NK cells in patients with iron overload are known to have more intracellular reactive oxygen species (ROS) [62].

The effects of vitamin C on NK cell cytotoxicity against ovarian cancer cells was also studied in vivo in AA deficient mice. *Gulo*^−^/^−^ mice that are dependent on dietary AA were not supplemented for 2 weeks and sub sequentially inoculated with MOSECs (murine ovarian surface epithelial cells) and compared with *Gulo*^−^/^−^ mice that received normal AA supplementation. After the inoculation of tumour cells, all animals received normal AA supplementation. *Gulo*^−^/^−^ mice that were AA-depleted during this period survived shorter than supplemented mice. NK cells isolated from these AA-depleted mice showed a significant decrease in killing capacity in vitro compared to AA-supplemented mice and wildtype mice. In concordance, their NK cells showed reduced expression of the activating receptors CD69 and NKG2D. Furthermore, these NK cells produced less IFN-γ and displayed reduced production of the cytolytic proteins perforin and granzyme B [63].

In conclusion, AA might have different effects on NK cells during different stages. Early and late human NK cell development is enhanced by AA in vitro, however the effect in vivo remains to be shown. It is likely that the effect of AA at this time point is caused by its role as an epigenetic regulator, since this is also observed in T cells that share various developmental steps.

The influence of AA on NK cell function is not fully determined yet. In most in vitro studies in which NK cells of healthy volunteers (that probably have normal AA levels) were used, no effect of AA was observed. However, in AA-deficient mice, NK cell function was decreased compared to mice with normal levels of AA. Furthermore, in two human studies employing NK cells with an impaired function AA was able to restore NK cell function to almost normal. These results suggest that at least physiological levels of AA are necessary for normal NK cell function and AA is probably not able to increase the cytotoxicity of NK cells that function normally but can help to restore the function in NK cells that are impaired.

It is unknown whether next to NK cells, the development and function of recently identified other members of the ILC family are also enhanced by AA. This may be important, because other ILC subsets may also provide immunity while T cell immunity has not yet recovered. Furthermore, it has been shown that, in allogeneic HSCT higher ILC 3 numbers are associated with less GVHD [64].

## 5. Conclusions

**Figure 1:**
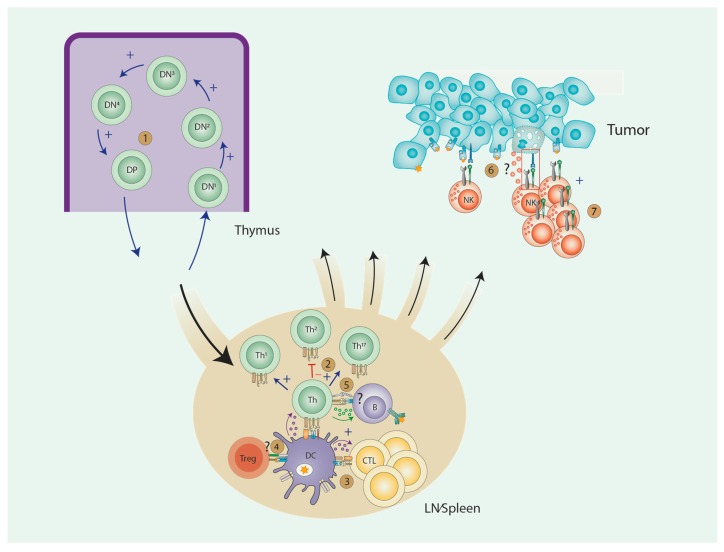
Effects of AA on Immune cells. **1**. T cell development: Enhanced T cell development due to fast transition from DN to DD stage. **2**. Th cell differentiation: skewing towards Th1 and Th17, with inhibition of Th2 polarization. **3**. CTL induction: Increased induction of CTLs due to production of IL-15 and IL-12 by DCs. **4**. Treg induction: Current data are conflicting. **5**. B cell: No conclusive data. **6**. NK cell function: No conclusive data. **7**. NK cell proliferation: increased NK cell proliferation.

AA has multiple effects on the development, proliferation and function of lymphocytes. An overview of these effects and the relationship between different cell types can be seen in Figure 1. T-lymphocytes have been most extensively studied in this context: AA positively influences T cell development and maturation, especially in case of AA deficiency. There is very limited and conflicting data on the effects of AA on B-lymphocyte biology. As for NK cells, AA positively influences NK cell proliferation but its role in NK cell function is less clear. A limited number of studies suggest that NK cell function required normal AA levels, while supraphysiological levels do not enhance NK cell function. Overall, most conclusions are based on in vitro studies, that are difficult to interpret and compare since there are many differences in experimental setups (multiple derivates of AA used in various concentrations and different incubation times). AA is also known to oxidate easily to dehydroascorbate in cell-cultures. The in vivo studies require careful interpretation as well: little data is available on local (intracellular) AA levels, while it is known that intracellular AA levels can be more than 1000-fold higher compared to plasma levels.

The studies discussed in the present review provide some insight in the mechanisms that underpin the effect on AA on lymphocytes. There are important indications that AA acts as an epigenetic regulator/cofactor in TET-mediated DNA and histone demethylation. Plausibly, AA’s epigenetic functions are mostly seen in cells that undergo change (e.g., early T cell development, T helper cell differentiation). In situations of cells under stress (e.g., thalassemia, sepsis), the antioxidant properties of AA are probably more important.

Since AA is a cheap supplement with limited side-effects, it is worthwhile to speculate on its potential for cancer patients that are proven to have lower serum AA levels. Here, AA could enhance immune reconstitution after treatments that give long immunosuppression (for instance, patients receiving intensive chemotherapy for leukaemia or autologous HSCT) as most studies indicate a positive effect of AA supplementation on lymphocyte development. In this case, AA’s effect on NK cells may be most significant, because NK cell reconstitution after myeloablative chemotherapy and HSCT is much faster than T cell reconstitution and could provide temporary immunity against infections [65]. Furthermore, NK cells are capable of recognizing and eliminating cancer cells. Currently, several clinical trials already study the anti-cancer potential of ex vivo generated NK cells. AA supplementation can be used to generate these NK cells in vitro but could also have an effect in vivo in the proliferation and survival of these cells. Furthermore, AA supplementation could may also positively influence T cell reconstitution after myeloablative therapy. It is possible that slow T cell regeneration is (partly) due to the AA-deficient state in these patients.

On the other hand, AA supplementation in cancer patients may also have negative effects. Most of the curative effect of an allogeneic hematopoietic stem cell transplantation is attributed to the graft versus tumour effect mediated by the donor T cells that recognize cancer cells. This effect could potentially be diminished by increasing the amount and function of Tregs by vitamin C, as seen in some studies [33,34]. On the other hand, another study shows decreased Treg activity following AA administration [41]. In addition, stimulation of Tregs may also protect against GVHD.

Thus, AA plays a multitude of roles in lymphocyte development and function. However, its exact mechanism(s) of action and its effects in human health and disease are currently unknown. Given its safe profile and the fact that most animal studies have important limitations, we currently prepare a single arm phase II study to test the safety (GVHD) and efficacy of AA supplementation after allogeneic stem cell transplantation. This study is likely to provide important insights in how this vitamin functions in a complex, diseased organism and what groups of patients may benefit from this safe supplement.

## References

[1] Cameron E., Pauling L. (1976). Supplemental ascorbate in the supportive treatment of cancer: Prolongation of survival times in terminal human cancer. Proc. Natl. Acad. Sci. USA.

[2] Creagan E.T., Moertel C.G., O’Fallon J.R., Schutt A.J., O’Connell M.J., Rubin J., Frytak S. (1979). Failure of high-dose vitamin C (ascorbic acid) therapy to benefit patients with advanced cancer. A controlled trial. N. Engl. J. Med..

[3] Moertel C.G., Fleming T.R., Creagan E.T., Rubin J., O’Connell M.J., Ames M.M. (1985). High-dose vitamin C versus placebo in the treatment of patients with advanced cancer who have had no prior chemotherapy. A randomized double-blind comparison. N. Engl. J. Med..

[4] Young J.I., Zuchner S., Wang G. (2015). Regulation of the Epigenome by Vitamin C. Annu. Rev. Nutr..

[5] Harrison F.E., Meredith M.E., Dawes S.M., Saskowski J.L., May J.M. (2010). Low ascorbic acid and increased oxidative stress in gulo(^−^/^−^) mice during development. Brain Res..

[6] Uchio R., Hirose Y., Murosaki S., Yamamoto Y., Ishigami A. (2015). High dietary intake of vitamin C suppresses age-related thymic atrophy and contributes to the maintenance of immune cells in vitamin C-deficient senescence marker protein-30 knockout mice. Br. J. Nutr..

[7] Carr A.C., Maggini S. (2017). Vitamin C and Immune Function. Nutrients.

[8] Wilson J.X. (2005). Regulation of vitamin C transport. Annu. Rev. Nutr..

[9] Omaye S.T., Schaus E.E., Kutnink M.A., Hawkes W.C. (1987). Measurement of vitamin C in blood components by high-performance liquid chromatography. Implication in assessing vitamin C status. Ann. N. Y. Acad. Sci..

[10] Evans R.M., Currie L., Campbell A. (1982). The distribution of ascorbic acid between various cellular components of blood, in normal individuals, and its relation to the plasma concentration. Br. J. Nutr..

[11] Roux E., Dumont-Girard F., Starobinski M., Siegrist C.A., Helg C., Chapuis B., Roosnek E. (2000). Recovery of immune reactivity after T-cell-depleted bone marrow transplantation depends on thymic activity. Blood.

[12] Bosch M., Khan F.M., Storek J. (2012). Immune reconstitution after hematopoietic cell transplantation. Curr. Opin. Hematol..

[13] Huijskens M.J., Walczak M., Koller N., Briede J.J., Senden-Gijsbers B.L., Schnijderberg M.C., Bos G.M., Germeraad W.T. (2014). Technical advance: Ascorbic acid induces development of double-positive T cells from human hematopoietic stem cells in the absence of stromal cells. J. Leukoc. Biol..

[14] Huijskens M.J., Walczak M., Sarkar S., Atrafi F., Senden-Gijsbers B.L., Tilanus M.G., Bos G.M., Wieten L., Germeraad W.T. (2015). Ascorbic acid promotes proliferation of natural killer cell populations in culture systems applicable for natural killer cell therapy. Cytotherapy.

[15] Huijskens M.J., Wodzig W.K., Walczak M., Germeraad W.T., Bos G.M. (2016). Ascorbic acid serum levels are reduced in patients with hematological malignancies. Results Immunol..

[16] Jenkinson E.J., Anderson G., Owen J.J. (1992). Studies on T cell maturation on defined thymic stromal cell populations in vitro. J. Exp. Med..

[17] Schmitt T.M., Zuniga-Pflucker J.C. (2002). Induction of T cell development from hematopoietic progenitor cells by delta-like-1 in vitro. Immunity.

[18] Meek B., Cloosen S., Borsotti C., Van Elssen C.H., Vanderlocht J., Schnijderberg M.C., van der Poel M.W., Leewis B., Hesselink R., Manz M.G. (2010). In vitro-differentiated T/natural killer-cell progenitors derived from human CD34^+^ cells mature in the thymus. Blood.

[19] Manning J., Mitchell B., Appadurai D.A., Shakya A., Pierce L.J., Wang H., Nganga V., Swanson P.C., May J.M., Tantin D. (2013). Vitamin C promotes maturation of T-cells. Antioxid. Redox Signal..

[20] Campbell J.D., Cole M., Bunditrutavorn B., Vella A.T. (1999). Ascorbic acid is a potent inhibitor of various forms of T cell apoptosis. Cell. Immunol..

[21] Maeng H.G., Lim H., Jeong Y.J., Woo A., Kang J.S., Lee W.J., Hwang Y.I. (2009). Vitamin C enters mouse T cells as dehydroascorbic acid in vitro and does not recapitulate in vivo vitamin C effects. Immunobiology.

[22] Eylar E., Baez I., Navas J., Mercado C. (1996). Sustained levels of ascorbic acid are toxic and immunosuppressive for human T cells. P. R. Health Sci. J..

[23] Hong J.M., Kim J.H., Kang J.S., Lee W.J., Hwang Y.I. (2016). Vitamin C is taken up by human T cells via sodium-dependent vitamin C transporter 2 (SVCT2) and exerts inhibitory effects on the activation of these cells in vitro. Anat. Cell Biol..

[24] Fraser R.C., Pavlovic S., Kurahara C.G., Murata A., Peterson N.S., Taylor K.B., Feigen G.A. (1980). The effect of variations in vitamin C intake on the cellular immune response of guinea pigs. Am. J. Clin. Nutr..

[25] Badr G., Bashandy S., Ebaid H., Mohany M., Sayed D. (2012). Vitamin C supplementation reconstitutes polyfunctional T cells in streptozotocin-induced diabetic rats. Eur. J. Nutr..

[26] Kennes B., Dumont I., Brohee D., Hubert C., Neve P. (1983). Effect of vitamin C supplements on cell-mediated immunity in old people. Gerontology.

[27] Kay N.E., Holloway D.E., Hutton S.W., Bone N.D., Duane W.C. (1982). Human T-cell function in experimental ascorbic acid deficiency and spontaneous scurvy. Am. J. Clin. Nutr..

[28] Noh K., Lim H., Moon S.K., Kang J.S., Lee W.J., Lee D., Hwang Y.I. (2005). Mega-dose Vitamin C modulates T cell functions in Balb/c mice only when administered during T cell activation. Immunol. Lett..

[29] Chang H.-H., Chen C., Lin J.-Y. (2009). High dose vitamin C supplementation increases the Th1/Th2 cytokine secretion ratio, but decreases eosinophilic infiltration in bronchoalveolar lavage fluid of ovalbumin-sensitized and challenged mice. J. Agric. Food Chem..

[30] Jeong Y.J., Hong S.W., Kim J.H., Jin D.H., Kang J.S., Lee W.J., Hwang Y.I. (2011). Vitamin C-treated murine bone marrow-derived dendritic cells preferentially drive naive T cells into Th1 cells by increased IL-12 secretions. Cell. Immunol..

[31] Song M.H., Nair V.S., Oh K.I. (2017). Vitamin C enhances the expression of IL17 in a Jmjd2-dependent manner. BMB Rep..

[32] Jeong Y.J., Kim J.H., Hong J.M., Kang J.S., Kim H.R., Lee W.J., Hwang Y.I. (2014). Vitamin C treatment of mouse bone marrow-derived dendritic cells enhanced CD8(+) memory T cell production capacity of these cells in vivo. Immunobiology.

[33] Sasidharan Nair V., Song M.H., Oh K.I. (2016). Vitamin C Facilitates Demethylation of the Foxp3 Enhancer in a Tet-Dependent Manner. J. Immunol..

[34] Yue X., Trifari S., Aijo T., Tsagaratou A., Pastor W.A., Zepeda-Martinez J.A., Lio C.W., Li X., Huang Y., Vijayanand P. (2016). Control of Foxp3 stability through modulation of TET activity. J. Exp. Med..

[35] Tahiliani M., Koh K.P., Shen Y., Pastor W.A., Bandukwala H., Brudno Y., Agarwal S., Iyer L.M., Liu D.R., Aravind L. (2009). Conversion of 5-methylcytosine to 5-hydroxymethylcytosine in mammalian DNA by MLL partner TET1. Science.

[36] Ito S., D’Alessio A.C., Taranova O.V., Hong K., Sowers L.C., Zhang Y. (2010). Role of Tet proteins in 5mC to 5hmC conversion, ES-cell self-renewal and inner cell mass specification. Nature.

[37] Blaschke K., Ebata K.T., Karimi M.M., Zepeda-Martinez J.A., Goyal P., Mahapatra S., Tam A., Laird D.J., Hirst M., Rao A. (2013). Vitamin C induces Tet-dependent DNA demethylation and a blastocyst-like state in ES cells. Nature.

[38] Chung T.L., Brena R.M., Kolle G., Grimmond S.M., Berman B.P., Laird P.W., Pera M.F., Wolvetang E.J. (2010). Vitamin C promotes widespread yet specific DNA demethylation of the epigenome in human embryonic stem cells. Stem Cells.

[39] Nikolouli E., Hardtke-Wolenski M., Hapke M., Beckstette M., Geffers R., Floess S., Jaeckel E., Huehn J. (2017). Alloantigen-Induced Regulatory T Cells Generated in Presence of Vitamin C Display Enhanced Stability of Foxp3 Expression and Promote Skin Allograft Acceptance. Front. Immunol..

[40] Kasahara H., Kondo T., Nakatsukasa H., Chikuma S., Ito M., Ando M., Kurebayashi Y., Sekiya T., Yamada T., Okamoto S. (2017). Generation of allo-antigen-specific induced Treg stabilized by vitamin C treatment and its application for prevention of acute graft versus host disease model. Int. Immunol..

[41] Gao Y., Lu B., Zhai J., Liu Y., Qi H., Yao Y., Chai Y., Shou S. (2017). The Parenteral Vitamin C Improves Sepsis and Sepsis-Induced Multiple Organ Dysfunction Syndrome via Preventing Cellular Immunosuppression. Mediat. Inflamm..

[42] Ichiyama K., Mitsuzumi H., Zhong M., Tai A., Tsuchioka A., Kawai S., Yamamoto I., Gohda E. (2009). Promotion of IL-4- and IL-5-dependent differentiation of anti-μ-primed B cells by ascorbic acid 2-glucoside. Immunol. Lett..

[43] Woo A., Kim J.H., Jeong Y.J., Maeng H.G., Lee Y.T., Kang J.S., Lee W.J., Hwang Y.I. (2010). Vitamin C acts indirectly to modulate isotype switching in mouse B cells. Anat. Cell Biol..

[44] Tanaka M., Muto N., Gohda E., Yamamoto I. (1994). Enhancement by ascorbic acid 2-glucoside or repeated additions of ascorbate of mitogen-induced IgM and IgG productions by human peripheral blood lymphocytes. Jpn. J. Pharmacol..

[45] Prinz W., Bloch J., Gilich G., Mitchell G. (1980). A systematic study of the effect of vitamin C supplementation on the humoral immune response in ascorbate-dependent mammals. I. The antibody response to sheep red blood cells (a T-dependent antigen) in guinea pigs. Int. J. Vitam. Nutr. Res..

[46] Feigen G.A., Smith B.H., Dix C.E., Flynn C.J., Peterson N.S., Rosenberg L.T., Pavlovic S., Leibovitz B. (1982). Enhancement of antibody production and protection against systemic anaphylaxis by large doses of vitamin C. Res. Commun. Chem. Pathol. Pharmacol..

[47] Albers R., Bol M., Bleumink R., Willems A.A., Pieters R.H. (2003). Effects of supplementation with vitamins A, C, and E, selenium, and zinc on immune function in a murine sensitization model. Nutrition.

[48] Hesta M., Ottermans C., Krammer-Lukas S., Zentek J., Hellweg P., Buyse J., Janssens G.P. (2009). The effect of vitamin C supplementation in healthy dogs on antioxidative capacity and immune parameters. J. Anim. Physiol. Anim. Nutr..

[49] Goodwin J.S., Garry P.J. (1983). Relationship between megadose vitamin supplementation and immunological function in a healthy elderly population. Clin. Exp. Immunol..

[50] Amakye-Anim J., Lin T.L., Hester P.Y., Thiagarajan D., Watkins B.A., Wu C.C. (2000). Ascorbic acid supplementation improved antibody response to infectious bursal disease vaccination in chickens. Poult. Sci..

[51] Wu C.C., Dorairajan T., Lin T.L. (2000). Effect of ascorbic acid supplementation on the immune response of chickens vaccinated and challenged with infectious bursal disease virus. Vet. Immunol. Immunopathol..

[52] Vallance S. (1977). Relationships between ascorbic acid and serum proteins of the immune system. Br. Med. J..

[53] Prinz W., Bortz R., Bregin B., Hersch M. (1977). The effect of ascorbic acid supplementation on some parameters of the human immunological defence system. Int. J. Vitam. Nutr. Res..

[54] Anderson R., Oosthuizen R., Maritz R., Theron A., Van Rensburg A.J. (1980). The effects of increasing weekly doses of ascorbate on certain cellular and humoral immune functions in normal volunteers. Am. J. Clin. Nutr..

[55] Huwyler T., Hirt A., Morell A. (1985). Effect of ascorbic acid on human natural killer cells. Immunol. Lett..

[56] Toliopoulos I.K., Simos Y.V., Daskalou T.A., Verginadis I.I., Evangelou A.M., Karkabounas S.C. (2011). Inhibition of platelet aggregation and immunomodulation of NK lymphocytes by administration of ascorbic acid. Indian J. Exp. Biol..

[57] Vojdani A., Ghoneum M. (1993). In vivo effect of ascorbic acid on enhancement of natural killer cell activity. Nutr. Res..

[58] Heuser G., Vojdani A. (1997). Enhancement of natural killer cell activity and T and B cell function by buffered vitamin C in patients exposed to toxic chemicals: The role of protein kinase-C. Immunopharmacol. Immunotoxicol..

[59] Farmakis D., Giakoumis A., Polymeropoulos E., Aessopos A. (2003). Pathogenetic aspects of immune deficiency associated with beta-thalassemia. Med. Sci. Monit..

[60] Atasever B., Ertan N.Z., Erdem-Kuruca S., Karakas Z. (2006). In vitro effects of vitamin C and selenium on NK activity of patients with beta-thalassemia major. Pediatr. Hematol. Oncol..

[61] Lynch S.R., Cook J.D. (1980). Interaction of vitamin C and iron. Ann. N. Y. Acad. Sci..

[62] Hua Y., Wang C., Jiang H., Wang Y., Liu C., Li L., Liu H., Shao Z., Fu R. (2017). Iron overload may promote alteration of NK cells and hematopoietic stem/progenitor cells by JNK and P38 pathway in myelodysplastic syndromes. Int. J. Hematol..

[63] Kim J.E., Cho H.S., Yang H.S., Jung D.J., Hong S.W., Hung C.F., Lee W.J., Kim D. (2012). Depletion of ascorbic acid impairs NK cell activity against ovarian cancer in a mouse model. Immunobiology.

[64] Munneke J.M., Bjorklund A.T., Mjosberg J.M., Garming-Legert K., Bernink J.H., Blom B., Huisman C., van Oers M.H., Spits H., Malmberg K.J. (2014). Activated innate lymphoid cells are associated with a reduced susceptibility to graft-versus-host disease. Blood.

[65] Vacca P., Montaldo E., Croxatto D., Moretta F., Bertaina A., Vitale C., Locatelli F., Mingari M.C., Moretta L. (2016). NK Cells and Other Innate Lymphoid Cells in Hematopoietic Stem Cell Transplantation. Front. Immunol..

